# Arthritic role of *Porphyromonas gingivalis* in collagen-induced arthritis mice

**DOI:** 10.1371/journal.pone.0188698

**Published:** 2017-11-30

**Authors:** Hyerin Jung, Seung Min Jung, Yeri Alice Rim, Narae Park, Yoojun Nam, Jennifer Lee, Sung-Hwan Park, Ji Hyeon Ju

**Affiliations:** 1 Catholic iPSC Research Center, College of Medicine, The Catholic University of Korea, Seoul, Republic of Korea; 2 Division of Rheumatology, Department of Internal Medicine, Seoul St. Mary's Hospital, College of Medicine, The Catholic University of Korea, Seoul, Republic of Korea; 3 Division of Rheumatology, Department of Internal Medicine, College of Medicine, Yonsei University, Seoul, Republic of Korea; US Department of Veterans Affairs, UNITED STATES

## Abstract

Epidemiological studies show an association between rheumatoid arthritis (RA) and periodontal disease. *Porphyromonas gingivalis* (*P*.*gingivalis*) is a well-known pathogen in periodontitis. This study investigated the pathogenic effects of *P*.*gingivalis* on autoimmune arthritis *in vivo*. Collagen-induced arthritis (CIA) mice were intraperitoneally injected with W83 and 2561 strains of *P*.*gingivalis*. Infection with *P*.*gingivalis* exacerbated arthritis score in CIA mice. Synovial inflammation and bone destruction in CIA mice infected with *P*.*gingivalis* were more severe than in uninfected CIA mice. Both W83 and 2561 strains were more pro-arthritic after arthritis symptom was fully activated. Interestingly, only W83 strain was arthritogenic before autoimmune reaction initiated. Citrullination was detected in synovial tissue of CIA mice and CIA mice inoculated with *P*.*gingivalis*, but not in normal control mice. The citrullinated area was greater in *P*.*gingivalis*-infected CIA mice than in non-infected CIA mice. This study showed that *P*.*gingivalis* exacerbated disease in a mouse model of autoimmune arthritis and increased the expression of citrullinated antigens in the synovium. The arthritogenic effects of *P*.*gingivalis* were at least in part, dependent upon the bacterial strain with or without fimbriae expression, route and time of infection. *P*.*gingivalis*-mediated citrullination may explain the possible link between periodontal disease and RA.

## Introduction

Rheumatoid arthritis (RA) is a systemic autoimmune disease characterized by joint inflammation and destruction [1]. Production of rheumatoid factor and autoantibodies leads to increased functional disability and morbidity [2]. Although the exact pathophysiology of RA is unclear, it does involve a combination of genetic and environmental factors [3–5].

Citrullination is an important post-translational modification that is ubiquitous in normal physiology. It is a process which arginine is conversed into citrulline in proteins. Peptidylarginine deaminase (PAD) is the enzyme that is responsible for this process [6,7]. PAD enzymes are normally involved in various regulatory processes in human [8,9]. Since PAD involves in biological processes such as epidermal differentiation, hair follicles maturation, and other epigenetic regulations, it is reported to be related to diseases such as psoriasis or multiple sclerosis [10]. PAD is also known to affect the insulation of nerve fibers which induce disease conditions such as alzheimer or prion diseases [8,10,11]. Also, citrullination of chemokines was recently shown to have functional roles in binding, signaling and proteolytic cleavage [12,13]. Citrullination also play a critical role in RA pathogenesis [14,15]. Increased citrullination is observed in the RA synovium, and antibodies against citrullinated peptides are generated by RA-associated autoimmune responses [16–18]. Previous studies suggest that PAD mediates citrullination of proteins in RA as well [6,7,19–21].

PAD is present in most organisms, yet, PAD expressed by *Porphyromonas gingivalis* (PPAD) is the only active form of bacterial PAD [22]. *Porphyromonas gingivalis* (*P*.*gingivalis*) is a gram-negative, anaerobic bacterium. *P*.*gingivalis* is usually found in oral cavities, which induce periodontal disease such as periodontitis [9]. The physiological role of PPAD is yet not known, however, it was suggested to enhance the bacterial survival by producing ammonia during deamination [23]. Ammonia neutralizes the acidic condition in the periodontal pocket and optimizes gingipain and PPAD function which induce ATP production and negatively regulate the neutrophil function [24].

Because of these characteristics, the relation between *P*.*gingivalis*, citrullination, and RA was actively investigated [9,25–27]. It is expected that when a host is infected with *P*.*gingivalis*, PPAD converts peptidyl-arginine into peptidyl-citrulline and T cells are activated by bacterial lipopolysaccharides. These T cells then activate B cells, leading to production of anti-citrullinated protein/peptide antibodies (ACPA) specific for citrullinated peptides. Citrullinated peptide-ACPA immune complexes are then formed and trigger inflammation in the joints of RA patients. During this process, citrullinated peptides originating from *P*.*gingivalis* and host peptides citrullinated by PPAD act as autoantigens that exacerbate autoimmune responses associated with RA [28–31]. Several isotopic forms of PAD in *P*.*gingivalis* and humans are reported [22,32,33]. PAD2 and PAD4 expression was shown in the rheumatoid synovium and synovial fluid cells [20,34,35]. PAD4 was present in the synovial fluid of RA patients and patients with spondyloathropathy or OA. On the other hand, PAD2 was expressed in the knee joint of RA patients, but not in OA patients [33]. It was also shown that the PAD expression in the synovium tissue correlated with infiltration of inflammatory cells, synovial thickening, and synovium vascularity [20].

Based on these observations, we aimed to examine the pathogenic role of *P*.*gingivalis* in autoimmune arthritis. The arthritogenic effects of *P*.*gingivalis* was confirmed by bacterial strain (i.e. W83 and 2561), route, and time point of inoculation. We conducted clinical and histological analyses of a collagen-induced arthritis (CIA) mouse model infected with *P*.*gingivalis*. CIA mice infected with *P*.*gingivalis* showed increased expression of enolase, fibronectin, and citrullinated antigens in the synovial region. This study may provide a possible link between *P*.*gingivalis* and RA.

## Materials and methods

### Ethical approval

All procedures involving animals were performed in accordance with the Laboratory Animals Welfare Act, the Guide for the Care and Use of Laboratory Animals, and the Guidelines and Policies for Rodent Experimentation provided by the Institutional Animal Care and Use Committee of the School of Medicine of The Catholic University of Korea. Experiments were performed according to the ARRIVE guidelines [9]. The study protocol was approved by the Institutional Review Board of The Catholic University of Korea (CUMC-2013-0011-02). Synovial samples from patients with RA were obtained from the Catholic Human Disease Sample Bank. Samples were donated anonymously; therefore, the requirement for consent was waived by the Institutional Review Board.

### Induction and assessment of collagen-induced arthritis

Mice were each subdivided into 6 groups. Each group was composed of 5 mice. Non-treated mice were used as normal control group. To induce CIA, mice were immunized intradermally using 2mg/mL Bovine type II collagen (CII; Chondrex, Redmond, WA, USA) emulsified 1:1 with 2mg/mL Complete Freund’s adjuvant (CFA; Chondrex, Redmond, WA, USA). 6-week-old male DBA/1J mice (Orient Bio, Seongnam, Korea) were intradermally injected at day 0 with 100mL of CII/CFA emulsion. CII emulsified 1:1 with Incomplete Freund’s adjuvant (IFA; Chondrex, Redmond, WA, USA) was injected into mice as a booster immunization 21 days after the first immunization.

Development of arthritis was monitored and scored in a blinded manner. Disease severity was scored three times a week. The severity of arthritis in each front and hind paw was scored from 0 to 4 (0, normal; 1, mild swelling confined to the tarsals; 2, swelling of two or more toes or joints, or increased swelling; 3, moderate swelling extending from the ankle to the metatarsal joints; and 4, severe swelling encompassing the ankle, foot, and digits). A representative arthritis score was determined by summing the scores of all four paws [36].

### *Bacterial* infection

*Porphyromonas gingivalis* W83 (ATCC BAA-308, VA, USA) and *Porphyromonas gingivalis* 2561 (Kyung Hee University, Seoul, Korea), *Fusobacterium nucleatum* (ATCC 25586, VA, USA) were used in this experiment. *P*.*gingivalis* strains W83, 2561 and *Fusobacterium nucleatum* (F.n) were grown in anaerobic conditions.

Oral infection group mice were infected before and after CIA induction. For oral infection, mice were inoculated with 1×10^9^ colony-forming units (CFU) of bacteria in 30 μl PBS with 2% carboxymethyl cellulose (Sigma-Aldrich, St. Louis, MO, USA). Bacteria were administered to the oral cavity of each mouse through a feeding needle two times a week.

For Intraperitoneal infection, mice were injected with 1×10^9^ CFU bacteria in a total volume of 100 μl. Intraperitoneal infection group mice were infected before and after CIA induction as well.

### Assessment of periodontitis

The total anaerobic bacteria count was determined using the McFarland turbidity standard protocol. Bacteria were directly swabbed from the gingival surface of CIA mice using a small sterilized brush, and then soaked in tryptic soy broth medium (Difco Laboratories, Detroit, MI, USA), and incubated in an anaerobic basket with an anaerobic gas pack (AnaeroGen, Oxoid Ltd., Hampshire, UK). Alveolar bone loss was examined morphometrically by measuring the distance between the cementoenamel junction (CEJ) and alveolar bone crest (ABC), as previously described [11,13]. Briefly, skulls were first autoclaved and defleshed, and the jaws were immersed in 3% hydrogen peroxide overnight and then stained with 1% methylene blue for 1 min. The distance from the CEJ to the ABC was assessed at five buccal sites per mouse using the Visiopharm Integrator System (Visiopharm, Horsholm, Denmark). All data were analyzed using GraphPad Prism 5 software (GraphPad, San Diego, CA, USA).

### Histological assessment

Mice were sacrificed by CO_2_ inhalation. The joint tissues from the hind paws were fixed in 4% paraformaldehyde and decalcified in 10% EDTA bone decalcifier prior to embedding in paraffin. Paraffin sections were then prepared (5μm thickness) and stained with hematoxylin and eosin, safranin O, and toluidine blue.

The inflammation score and joint destruction score were measured using the procedure of Huckel et al. by three individual researchers in a blinded manner [37,38]. Inflammation and destruction scores were determined microscopically by blinded examiners. The inflammation score of each group was measured by the severity of infiltration and pannus formation. The destruction score was measured based on the cartilage and bone destruction [39,40].

Tartrate-resistant acid phosphatase (TRAP) staining was performed using a commercial kit (Sigma, MO, USA), with hematoxylin as the counterstain. TRAP-positive cells with three or more nuclei were counted as osteoclasts.

### Immunostaining

To confirm the presence of citrullinated proteins, joint tissues were stained with anti-CCP (12G1) antibody (AR13-MA0001, Abfrontier, Seoul, Korea) [41]. Joint tissues were deparaffinized in xylene and then hydrated through a graded series of ethanol solutions. Endogenous peroxidase activity was blocked with 3% H_2_O_2_ prepared in PBS. Staining was performed using Mouse on Mouse Basic Kit. Tissue sections were incubated with Mouse on Mouse (M.O.M) mouse IgG blocking agent to block nonspecific antibody binding. Anti-CCP (12G1) antibody (AR13-MA0001, Abfrontier, 1:100) was diluted in M.O.M Diluent buffer and incubated overnight at 4°C. IgG isotype control antibody (5415S, Cell Signaling, MA, USA) was used to confirm nonspecific antibody binding IgG. The sections were then incubated with Biotinylated Anti-Mouse Ig Reagent and an avidin-biotin complex. Chromogenic reactions were visualized with DAB Peroxidase (HRP) Substrate Kit (SK-4100, Vector Lab, CA, USA). Sections were counterstained with hematoxylin.

To detect the synovial proteins (i.e. enolase, vimentin, and fibronectin), anti-vimentin antibody (sc7558, Santa Cruz, CA, USA), anti-fibronectin antibody (ab23750, Abcam, Cambrige, UK), anti-enolase antibody (sc15343, Santa Cruz) were diluted at a concentration of 1: 100 in 5% normal goat serum containing 1% PBA. Slides were incubated at 4°C overnight. The sections were then incubated with Biotinylated Anti-Mouse Ig Reagent and an avidin-biotin complex. Chromogenic reactions were visualized with DAB Peroxidase (HRP) Substrate Kit (SK-4100, Vector Lab, CA, USA). Sections were counterstained with hematoxylin.

The co-localization of vimentin, fibronectin, and enolase was confirmed by fluorescence staining. Staining was performed using M.O.M Fluorescein Kit (BMK-2201, Vector Lab). Tissue sections were incubated with M.O.M mouse IgG blocking Reagent. Anti-CCP (12G1) antibody was diluted at a concentration of 1:100 in M.O.M Diluent buffer and was incubated at 4°C overnight. Fluorescein Avidin DCS was diluted in PBS and incubated at RT for 10 min. For serum blocking, 10% normal goat serum (S-1000, Vector Lab) containing 1% PBA was incubated at RT for 30 minutes. Anti-vimentin antibody (sc7558, Santa Cruz, CA, USA), anti-fibronectin antibody (ab23750, Abcam, Cambrige, UK), anti-enolase antibody (sc15343, Santa Cruz) were diluted at a concentration of 1: 100 in 5% normal goat serum containing 1% PBA. Slides were incubated at 4°C overnight. Alexa Fluor 594 goat anti-rabbit IgG (H+L) antibody (A11037, molecular probe, Oregon, USA) was diluted at a concentration of 1:200 in PBS and incubated for 40 minutes at RT. Nucleus staining was performed using 4',6-diamidino-2-phenylindole (DAPI; 10236276001, ROCHE, Basel, Switzerland).

### Quantitative image analysis

Automated image analysis techniques were used to quantify the number of TRAP-positive cells. To quantify TRAP staining and IHC staining, slide labeled with TRAP and ACPA were scanned using the Leica SCN400 slide scanner (Leica SCN400, Wetzlar, Germany). Using TissuemorphDP version software (Visiopharm, Denmark), the percentage of surface area per osteoclast (≥3 nuclei) and then DAB surface area were quantified. Assays were performed in triplicate.

### Statistical analysis

All experiments were repeated three or more times. The results are shown as mean and standard error of the mean. Error bars represent the standard error of the mean. Statistical analysis was performed and graphs were drawn using GraphPad Prism 5 (GraphPad). The T-test was applied to analyse non-parametric quantitative datasets, and the one-tailed p-value was calculated. *, P<0.01; **, P<0.005; and ***, P<0.001 indicated statistical significance.

## Results

### Effects of *P*.*gingivalis* on severity of collagen-induced arthritis (CIA)

**Fig 1:**
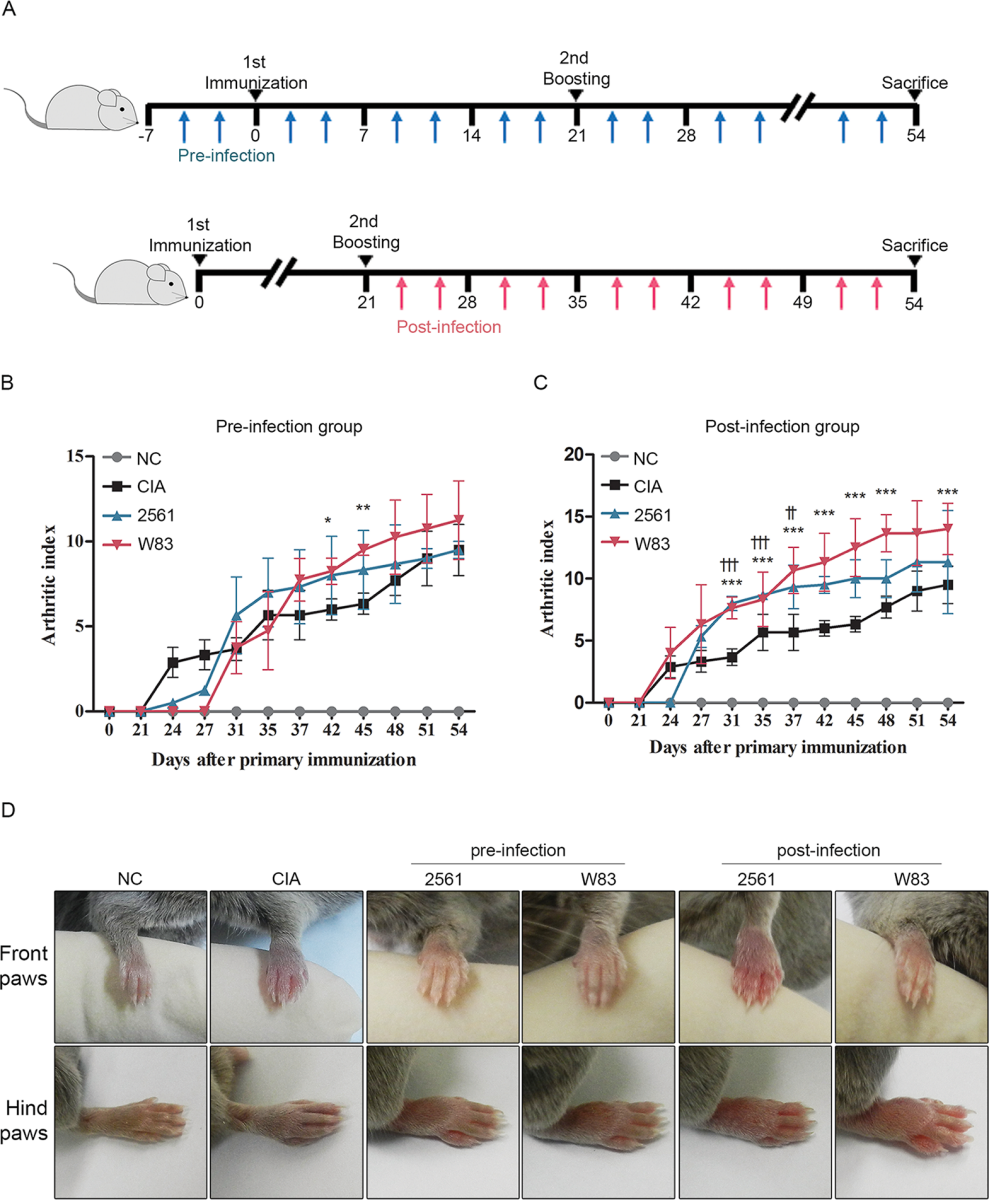
Effects of *P*.*gingivalis* on severity of collagen-induced arthritis (CIA). (A) Two *P*.*gingivalis* strains (W83 and 2561) were injected intraperitoneally into mice twice per week, starting either 1 week before the first immunization with collagen (Pre-infection group) or immediately after the second booster immunization (Post-infection group). Arthritis score of (B) pre-infected group and (C) post-infected group is shown. Arthritis scores were evaluated twice per week throughout the experimental period. The score for each paw ranged from 0 (no swelling) to 4 (erythema and severe swelling encompassing the ankle and foot); the scores for all four paws were summed to generate a representative arthritis score. Data represent the mean arthritis score (± standard error of the mean; SEM). ** P < 0.05 Pre-infection with W83 versus non-infected CIA; *** P < 0.01 Post-infection with W83 versus non-infected CIA; †† P < 0.05 Post-infection with 2561 versus non-infected CIA; ††† P < 0.01 Post-infection with 2561 versus non-infected CIA. (D) Representative images of front paws and hind paws from mice in each group.

CIA mice model is the most commonly studied autoimmune model of RA that show similar symptoms such as cartilage and bone destruction [42,43]. We investigated whether *P*.*gingivalis* infection affects the incidence or severity of CIA. *P*.*gingivalis* was inoculated orally and intraperitoneally with both W83 and 2561 strain. W83 is a strain lacking fimbriae expression, while 2561 is known to have several types of fimbriae on its membrane. To determine whether the time of infection affects CIA, we examined two different time points: before the first CII immunization (Pre-infection) and after the boosting CII immunization (Post-infection). Each group was infected twice a week with different strains of *P*.*gingivalis* (Fig 1A). When delivered orally, W83 showed increased arthritic index in the pre-infected time point (S 1A). The difference, however, was not observed in the post-infected group when orally delivered (S 1B). The inoculated mice showed defected gum regions than the normal control (NC) or CIA control (S 1C). This destruction was evaluated by methylene blue staining (S 1D). The length of the destructed cemantoenamel junction (CEJ)-alveolar bone crest (ABC) distance was increased in inoculated mice in both time points, which indicates successful induction of periodontitis (S 1E).

In the case of intraperitoneally inoculated mice, the W83 strain resulted in a higher arthritis score than 2561 in the Pre-infection setting (Fig 1B). Whereas in a Post-infection setting, the arthritis scores in mice infected with W83 or 2561 were higher than those in the non-infected CIA group (Fig 1C). After 1 week, all of the paws of CIA mice (infected and non-infected) were swollen, suggesting successful induction of arthritis. However, there was no difference between the two *P*.*gingivalis* strains in terms of severity of swelling (Fig 1D). To confirm if the symptoms are caused by PPAD, we compared the arthritic score of CIA mice infected with W83 or *Fusobacterium nucleatum* (F.n) (S 2). F.n is a bacterial strain with mutant PAD. The W83 infected CIA mice resulted in an increase in arthritis scores whereas the F.n infected CIA mice arthritis score was similar to that of CIA mice. Through this observation we can assume that the increased inflammation in the CIA mice paw was affected by PPAD.

### Histological analysis and osteoclastogenesis of arthritic joints from CIA mice treated with *P*.*gingivalis*

**Fig 2:**
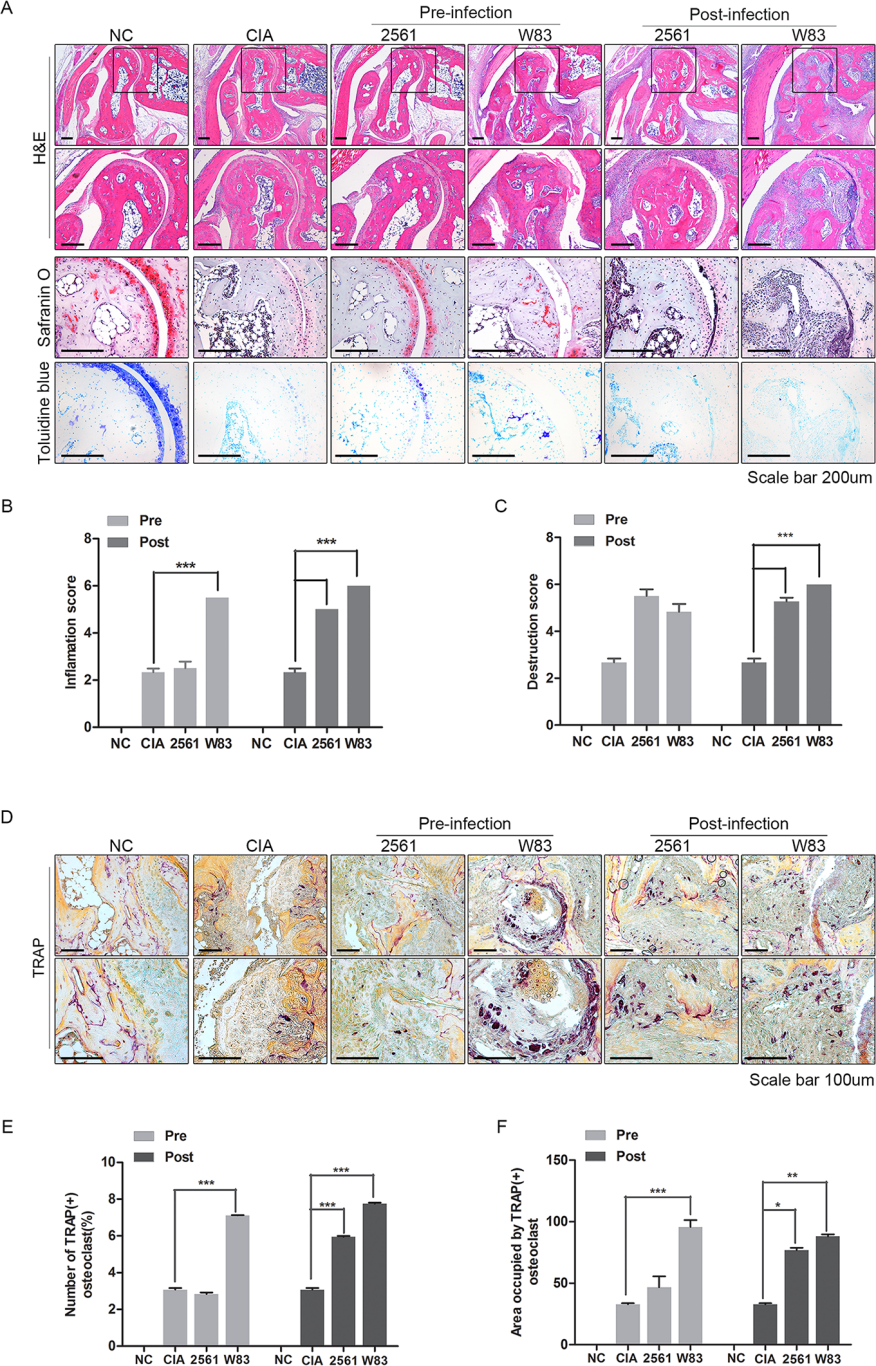
Histological analysis and osteoclastogenesis of arthritic joints from CIA mice infected with *P*.*gingivalis*. (A) Histological analysis of joints in the hind paws of animals from each group. Tissue sections were stained with hematoxylin and eosin, safranin O, and toluidine blue. Scale bars: 200 μm. (B) Inflammation scores and (C) joint destruction scores were calculated. Histological scores were evaluated by three independent observers in a blinded manner and expressed as the mean ± SEM (*p<0.05, **p<0.01, and ***p<0.001). (D) Osteoclastogenesis in synovial tissues was determined by staining with tartrate-resistant acid phosphatase (TRAP). Scale bar: 200 μm. (E) Area occupied by TRAP-positive osteoclasts. (F) TRAP-positive osteoclasts with multinucleated cells were counted using an automated system for quantitative image analysis. Data represent the mean of three independent counts (± SEM). *P < 0.05.

Since the significance was higher in intraperitoneally infected mice, we further analyzed the joint of mice inoculated by intraperitoneal injections. Infiltration by inflammatory cells, synovial proliferation, cartilage thinning, and joint destruction were observed in all CIA mice. However, *P*.*gingivalis* infection aggravated the pathology associated with CIA. Histology revealed that inflammatory cell infiltration and joint destruction in infected CIA mice was more severe than in CIA mice (Fig 2A). Safranin O and toluidine blue staining revealed cartilage thinning and erosion in the joints of all CIA mice. The erosive changes were more severe in infected CIA mice than in non-infected CIA mice. Using a semiquantitative scoring system, histological evaluation was performed. Inflammation (cell exudate or severe infiltration, synovial hyperplasia) and joint destruction (necrosis, erosion and pannus formation) appeared more severe in both the Pre-infected and Post-infected groups compared with non-infected CIA mice (Fig 2B and 2C). While, 2561 failed to affect the symptoms in the Pre-infected group (Fig 2B). The inflammation score was significantly higher in both the Pre-infected and Post-infected groups than in the non-infected CIA group. The destruction score tended to increase in 2561 and W83 Pre-infection groups compared with non-infected CIA group, but did not reach statistical significance. However, the destruction score did increase in the 2561 and W83 Post-infected groups (Fig 2C). Next, we examined the osteoclastogenic activity in each group using tartrate-resistant acid phosphatase (TRAP) staining. Osteoclastogenesis was observed in inflammatory synovium of all CIA mice except for NC mice (Fig 2D). The joint tissues of Pre-infected group and Post-infected group harbored more TRAP-positive cells than those of non-infected CIA mice. TRAP-positive osteoclasts with multinucleated cells were counted using an automated system for quantitative image analysis. TRAP-positive cells were increased in W83 inoculated CIA mice in the Pre-infected group (Fig 2E). Yet, in the Post-infected group, both stains increased the population of osteoclasts. The surface area of stained regions showed similar results (Fig 2F). In conclusion, we confirmed that W83 aggravate the arthritic symptoms in the Pre-infected group, however, both W83 and 2561 provoked arthritis in CIA mice. Also, *P*.*gingivalis* infection after immunization seemed to provoke the arthritis symptoms more efficiently.

### Citrullinated peptides are more common in CIA mice infected with *P*.*gingivalis*

**Fig 3:**
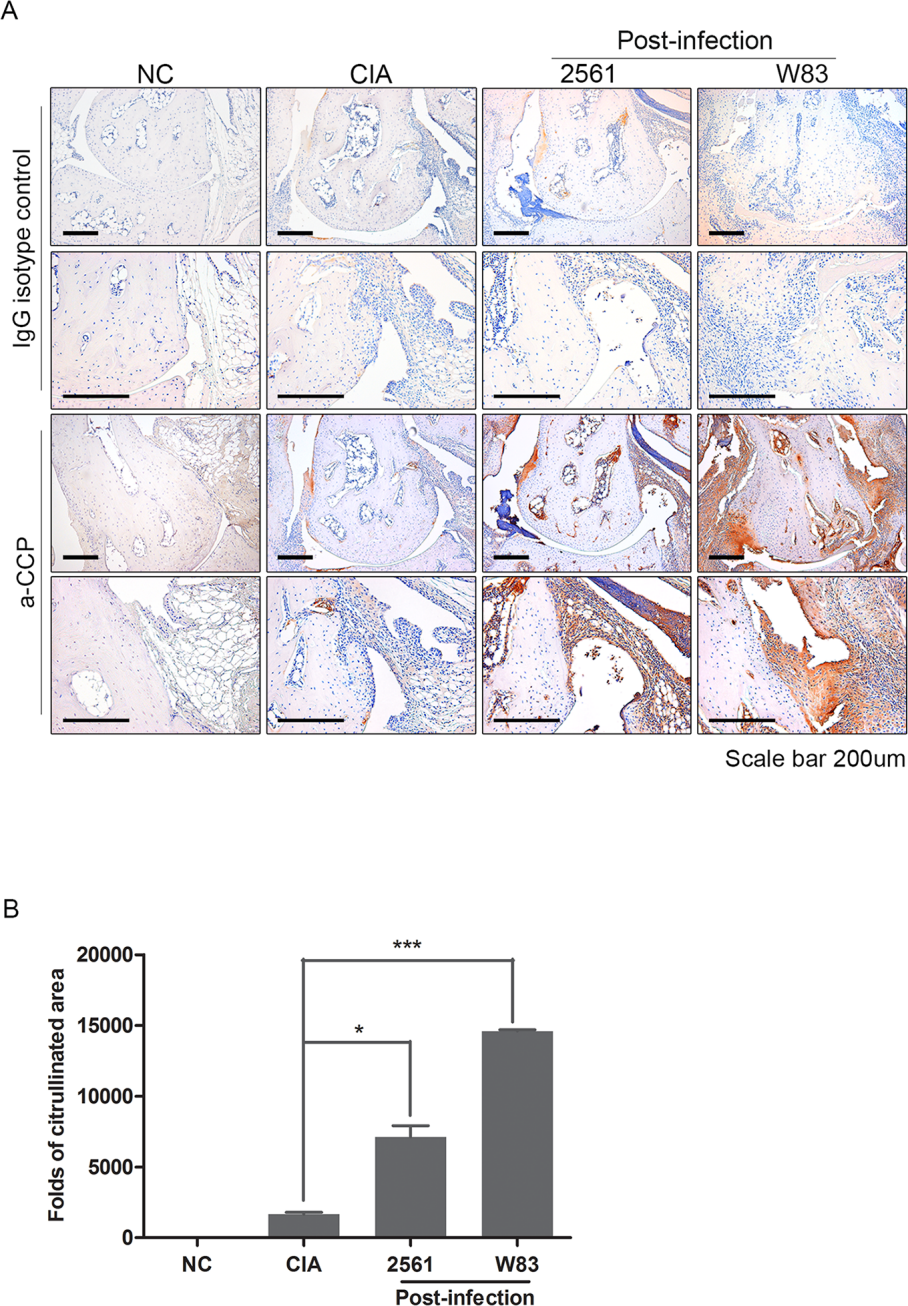
Expression of citrullinated peptides in the arthritic joints of mice with collagen-induced arthritis (CIA). An anti-citrullinated peptide antibody was produced by a hybridoma cell line derived by fusing myeloma cells with B cells from mice immunized with cyclic citrullinated peptide. (A) Immunohistochemical staining to confirm the presence of citrullinated peptides in the joints of normal control (NC) mice, non-infected CIA mice, and Post-infected CIA mice (n = 5 per group). The bottom row shows magnifications of the boxed areas. Scale bars: 200 μm. (B) Citrullinated regions were examined by quantitative image analysis. The fold increase in the size of the citrullinated area was then calculated relative to the same area in NC mice. Data represent the mean of three independent experiments (± standard error of the mean). *** P <0.001.

To feature the different effect of W83 and 2651 in arthritis, we further examined the Post-infected mice joint which seemed to be more affected by bacterial inoculation. By Immunohistochemical staining with anti-CCP antibody, we confirmed that citrullinated proteins were more extensively distributed in the joints of CIA mice infected with *P*.*gingivalis* than in non-infected CIA group (Fig 3A). Sections from the Pre-infected mice also stained positive for anti-CCP, but the intensity was much lower than that in the Post-infection group (data not shown). Automated image analysis was used to calculate the area of citrullination in W83 and 2561 Post-infection groups. The area of citrullination in Post-infection groups was significantly larger than that in non-infected CIA mice (Fig 3B). The staining results were more intense in mice inoculated with W83.

### Proteins targeted for citrullination are highly expressed in CIA mice infected with *P*.*gingivalis*

**Fig 4:**
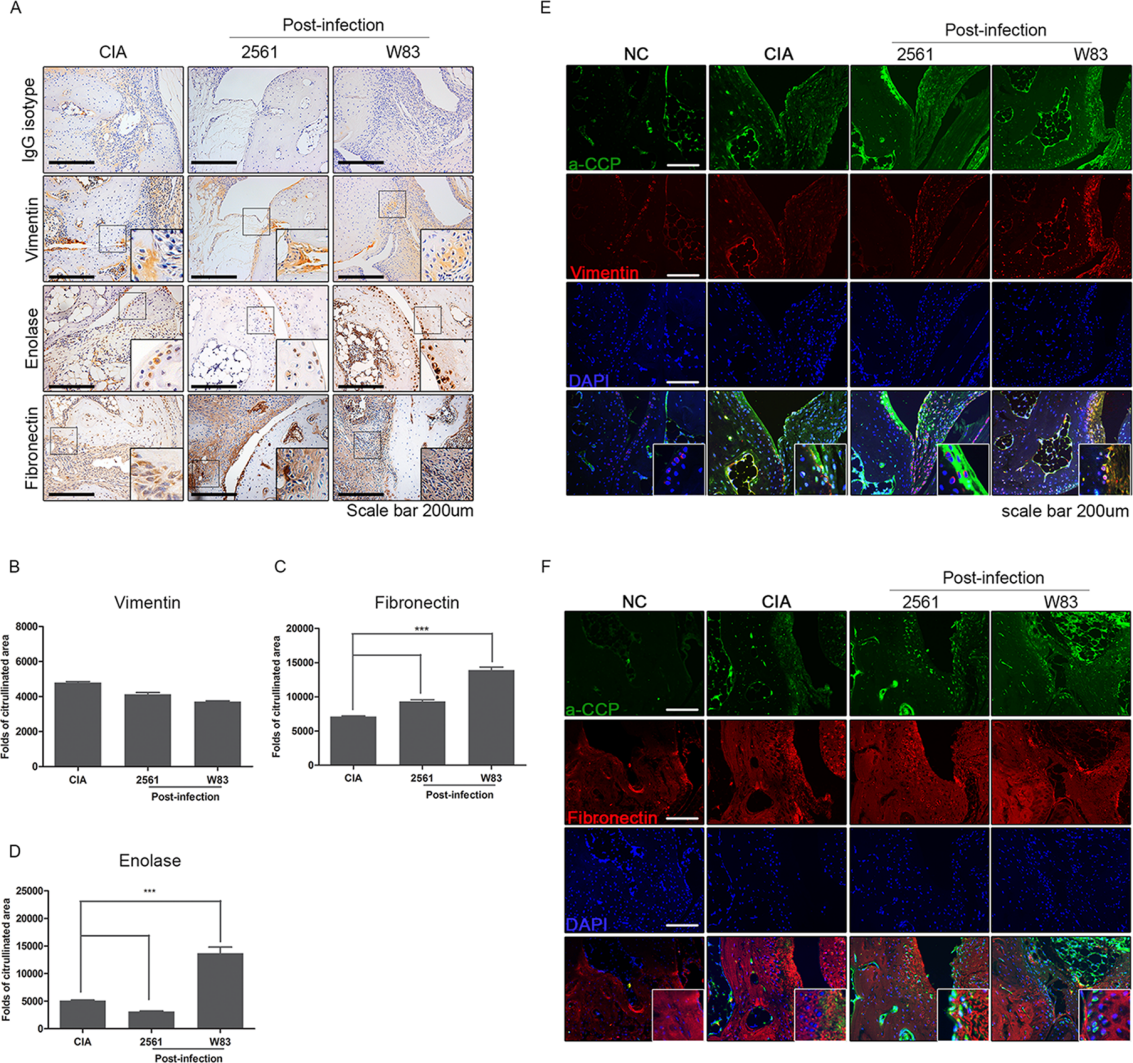
Citrullinated vimentin and fibronectin in synovial tissues of mice with collagen-induced arthritis (CIA) post-infected with *P*.*gingivalis*. (A) Immunohistochemical staining for vimentin, enolase, and fibronectin which are known to be prone to citrullination in the arthritic joints of CIA mice Post-infected with *P*.*gingivalis* (n = 3). The number of (B) vimentin-, (C) fibronectin-, and (D) enolase- positive cells was counted. The co-localization of citrulline with (E) vimentin and (F) fibronectin was confirmed. Data represent the mean ± SEM of three independent counts. ***p<0.001.

Expression of proteins (i.e. vimentin, enolase, and fibronectin) that are prone to citrullination in the joint tissue was examined. The expression of these proteins in the arthritic synovium of Post-infected groups was higher than that in non-infected CIA mice (Fig 4A). The area of these proteins in Post-infection groups was significantly larger than that in non-infected CIA mice. Vimentin, however, did not show any significant difference (Fig 4B). Fibronectin expression was increased in infected mice, especially with W83 (Fig 4C). Enolase was not increased in mice inoculated with 2561, yet, it was significantly increased in W83 infected mice (Fig 4D).

To confirm if these proteins were citrullinated, we detected the co-localization of citrulline and vimentin or fibronectin. Co-localization of citrulline peptides and vimentin were increased in all CIA induced mice (Fig 4E). The expression seemed to be highest in CIA mice infected with W83. This result was also shown in the case of fibronectin as well (Fig 4F). As a result, we have confirmed increased enolase, and fibronectin expression in the knee joint, and the increased co-localization with citrulline in infected mice.

## Discussion

The present study showed that *P*.*gingivalis* infection aggravated arthritis and increased citrullination in RA mouse model. Infection with *P*.*gingivalis* increased synovial inflammation in CIA mice, which is consistent with the findings of previous studies [44–46]. However, we found it interesting that citrullination of synovial peptides in CIA mice infected with *P*.*gingivalis* was higher than that in non-infected CIA mice. In this study, we demonstrated that *P*.*gingivalis* affects citrullination in the joint synovium *in vivo*.

Epidemiological studies consistently report an association between periodontal disease and RA; thus many researchers have attempted to identify a pathophysiological connection between the two diseases [9,25–27,47–49]. *P*.*gingivalis*, the causative agent of periodontitis, is an eligible candidate because it citrullinates peptides via bacterial PPAD. Citrullination is a critical process in RA pathogenesis because citrullinated peptides induce pro-inflammatory responses [50,51] and autoantibodies against citrullinated antigens increase osteoclastogenesis in RA [52]. Murine models of RA also exhibit citrullination of synovial proteins [53].

It is unclear how *P*.*gingivalis* promotes citrullination in the synovium; however, evidence suggests that the bacterium directly citrullinates the host proteins in RA. *P*.*gingivalis* DNA has been detected in the synovial fluid and tissues of patients with RA [54]. Moreover, PPAD targets human proteins *in vitro*, implying that *P*.*gingivalis* generates citrullinated antigens in RA patients. Marsez et al. reported that PPAD plays a critical role in the development and progression of CIA [45]. A recent study suggests that *P*.*gingivalis* induces autoimmune experimental arthritis via a mechanism that is independent of citrullination (e.g., induction of a Th17 response via toll-like receptor-2 and interleukin-1 signaling) [3]; thus further research is needed to clarify the arthritogenic role of *P*.*gingivalis*.

In this study, we inoculated CIA mice with two strains of *P*.*gingivalis* (i.e. 2561 and W83). W83 is afimbriated strain and 2561 is a fimbriated strain of *P*.*gingivalis*. The bacteria were delivered through two route; orally or intraperitoneally. Several studies showed significant results through oral infection of *P*.*gingivalis* [46,55–58]. Yet, results of intraperitoneally inoculated CIA mice showed more significant difference, especially between pre- and post- infection groups in our study (Fig 1B and 1C). We confirmed that W83 significantly provoked arthritis, yet, 2561 showed no difference compared to CIA control when inoculated before immunization (Fig 1B). When *P*.*gingivalis* was delivered after immunization, both strains showed increase arthritis score than that of CIA controls (Fig 1C). Fimbriae are reported to play a critical role in the adherence of *P*.*gingivalis* and in triggering the immune responses in the host [59]. The bond between the bacteria and the cell surface are usually mediated by non-specific forces such as covalent bones, Van der Waals forces, based on pH or electrostatics [60–62]. Attachment of fimbriae can be influenced by environmental factors such as pH, salinity, hydrophobicity and charge [63]. Through our observation, we suggest that the changed immune environment in CIA mice before or after immunization, might affect the attachment and pathogenesis of fimbriated strains of *P*.*gingivalis*.

Through our results, we confirmed increased expression of proteins prone to citrullination, and citrullinated peptides in these proteins. While the levels of vimentin was not changed, the expression of fibronectin and enolase was increased in W83 infected CIA mice (Fig 4B–4D). 2561 only affected the expression of fibronectin (Fig 4C). The increased citrullination of enolase by *P*.*gingivalis* agreed with the previous study reported by Wegner et al [22]. Increased expression of enolase is reported to be related to the progression of various diseases such as neuroblastoma and lung cancer [64–70]. Fibronectin is also reported as an agent associated with disease severity of both RA and periodontitis. Ghosh and colleagues reported that a proapoptotic fibronectin matrix increased the levels of nitric oxide and inducible nitric oxide synthase dose in the inflamed sites caused by bacteria [71]. This might suggest a clue to explain the increased expression of enolase and fibronectin with the provoked arthritic symptoms in infected CIA mice. Also this report can suggest a new connection between *P*.*gingivalis*, synovial proteins (i.e. enolase and fibronectin, and RA.

## Conclusions

The purpose of this experiment is to investigate the relationship between the severity of arthritis and the route or time point of infection with two strains of *P*.*gingivalis*. PAD expressed by *P*.*gingivalis* aggravated the disease symptoms in experimental arthritis model.

W83 showed more severe arthritis symptoms in Pre-treatment group compared to 2561 bacteria. However, in Post-treatment group, both of bacteria aggravated arthritis.

Bacterium infection in CIA mice increased several synovial protein (i.e. enolase and fibronectin) expression in the knee joint. Also, citrullinated forms of these proteins were increased in infected mice compared to that of CIA control mice.

Here, we showed that infection of CIA mice with the periodontal pathogen. *P*.*gingivalis* induced production of citrullinated antigens and aggravated both the clinical and histological signs of arthritis. Therefore, *P*.*gingivalis*-mediated citrullination may be the link between periodontal disease and RA.

## Supporting information

S1 Fig Oral infection of *P*.*gingivalis* confirm in CIA. (A, B) Arthritis severity score of NC mice (n = 5), CIA mice (n = 5), and CIA mice infected with *P*.*gingivalis* (n = 5). Pre and Post *P*.*gingivalis* were oral infected twice per week throughout the experimental period. The score for each paw ranged from 0 (no swelling) to 4 (erythema and severe swelling encompassing the ankle and foot); the scores for all four paws were summed to generate a representative arthritis score. (*, P<0.01; **, P<0.005; ***, P<0.001) (C) Representative image of jaw bone from mice in each group. (D) Methylene blue staining image of the jaw bone to examine the CEJ-ABC distance. (E) The distance was measured.
(TIF)

S2 Fig Inoculation with PAD-mutant bacteria. The arthritic index was compared between normal, CIA control mice and mice infected with W83 or *Fusobacterium nucleatum* (F.n).
(TIF)

S1 File ARRIVE guidelines checklist. Experiments were performed according to the ARRIVE guidelines.
(PDF)
