# Supplementary figures and images for: Arthritic role of Porphyromonas gingivalis in collagen-induced arthritis mice
Source: PLoS One. 2017 Nov 30;12(11):e0188698. doi: 10.1371/journal.pone.0188698 (PMC5708830; doi:10.1371/journal.pone.0188698)

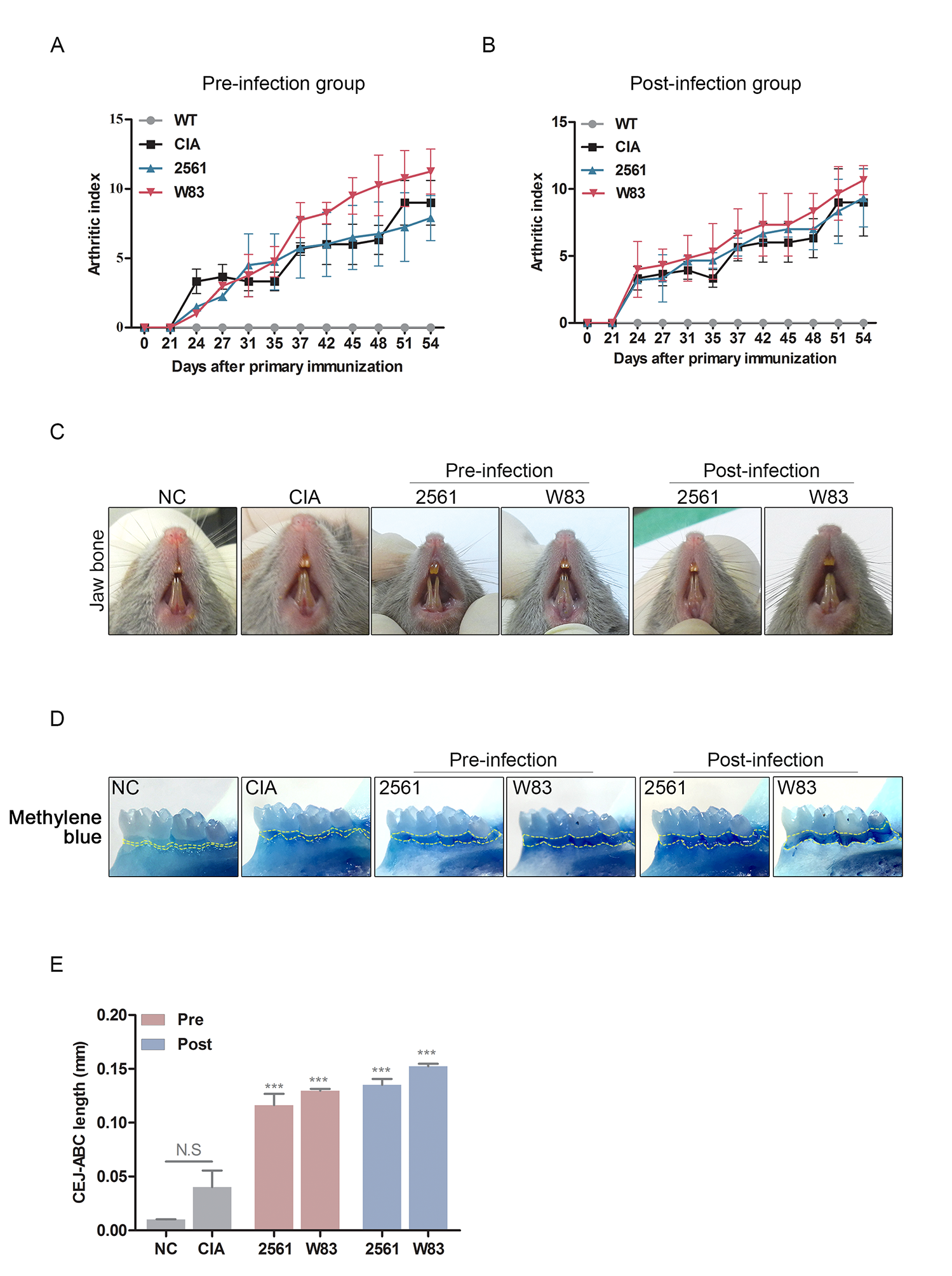

Supplement: S1 Fig — (A, B) Arthritis severity score of NC mice (n = 5), CIA mice (n = 5), and CIA mice infected with P.gingivalis (n = 5). Pre and Post P.gingivalis were oral infected twice per week throughout the experimental period. The score for each paw ranged from 0 (no swelling) to 4 (erythema and severe swelling encompassing the ankle and foot); the scores for all four paws were summed to generate a representative arthritis score. (*, P<0.01; **, P<0.005; ***, P<0.001) (C) Representative image of jaw bone from mice in each group. (D) Methylene blue staining image of the jaw bone to examine the CEJ-ABC distance. (E) The distance was measured. (TIF) [file pone.0188698.s001.tif]

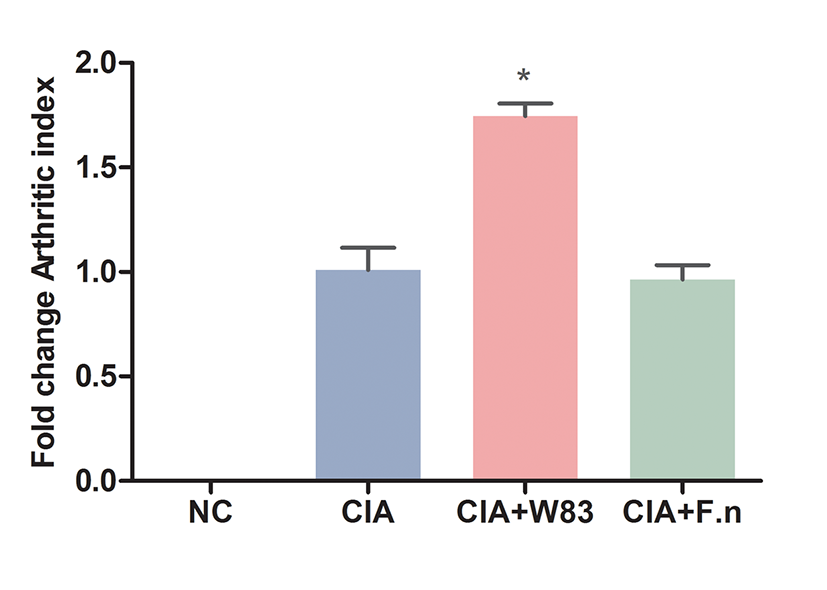

Supplement: S2 Fig — The arthritic index was compared between normal, CIA control mice and mice infected with W83 or Fusobacterium nucleatum (F.n). (TIF) [file pone.0188698.s002.tif]
